# Dissipation Dynamics of Doxycycline and Gatifloxacin and Accumulation of Heavy Metals during Broiler Manure Aerobic Composting

**DOI:** 10.3390/molecules26175225

**Published:** 2021-08-28

**Authors:** Lei Chu, Yongcui Wang, Bin Huang, Jian Ma, Xin Chen

**Affiliations:** 1 Key Laboratory of Pollution Ecology and Environment Engineering, Institute of Applied Ecology, Chinese Academy of Sciences, Shenyang 110016, China; chulei@alumni.purdue.edu (L.C.); huangbin@iae.ac.cn (B.H.); mroger@163.com (J.M.); 2 University of Chinese Academy of Sciences, Beijing 100039, China; 3 Wellhope Foods Co., Ltd., Shenyang 110164, China; 4 CAS Key Laboratory of Forest Ecology and Management, Institute of Applied Ecology, Chinese Academy of Sciences, Shenyang 110016, China; yongcuiwang@iae.ac.cn

**Keywords:** Doxycycline, Gatifloxacin, heavy metal, composting, degradation, accumulation

## Abstract

In this study, broilers were fed with heavy-metal-containing diets (Zn, Cu, Pb, Cr, As, Hg) at three rates (T1: 5 kg premix/ton feed, T2: 10 kg premix/ton feed and T3: 15 kg premix/ton feed) and Doxycycline (DOX) and Gatifloxacin (GAT) at low or high doses (T4: 31.2 mg DOX/bird/day and 78 mg GAT/bird/day, T5: 15.6 mg DOX/bird/day and 48 mg GAT/bird/day) to assess the accumulation of various heavy metals and the fate of two antibiotics in broiler manure after 35 days of aerobic composting. The results indicated that the two antibiotics changed quite differently during aerobic composting. About 14.96–15.84% of Doxycycline still remained at the end of composting, while Gatifloxacin was almost completely removed within 10 days of composting. The half-lives of Doxycycline were 13.75 and 15.86 days, while the half-lives of Gatifloxacin were only 1.32 and 1.38 days. Based on the Redundancy analysis (RDA), the concentration of antibiotics was significantly influenced by physico-chemical properties (mainly temperature and pH) throughout the composting process. Throughout the composting process, all heavy metal elements remained concentrated in organic fertilizer. In this study the Cr content reached 160.16 mg/kg, 223.98 mg/kg and 248.02 mg/kg with increasing premix feed rates, similar to Zn, which reached 258.2 mg/kg, 312.21 mg/kg and 333.68 mg/kg. Zn and Cr concentrations well exceeded the United States and the European soil requirements. This experiment showed that antibiotic residues and the accumulation of heavy metals may lead to soil contamination and pose a risk to the soil ecosystem.

## 1. Introduction

With the rapid development of animal husbandry in China, broiler chicken meat has become the second-largest meat industry after pork. However, in order to enhance the disease resistance of broilers and to promote rapid growth, antibiotics are often used in large quantities. Various trace elements are also often added in excessive amounts during the production process of broilers. In China, 150,000 tons to 200,000 tons of antibiotics have been used every year—or about 10 times the number in the United States [1]. About 70–90% of antibiotics and 60–70% of metals used in animal husbandry could be excreted in feces and urine without being metabolized by broilers [2].

Although most of the antibiotics that enter the soil can be degraded within less than 30 days, Roxithromycin, Opsarafloxacin, and Virginiamycin are still persistent [3]. Some antibiotics, such as Aureomycin, could be absorbed by food crops, such as vegetables and corn. Eating food contaminated with antibiotics can increase antibiotic resistance or lead to food poisoning and allergies. A study showed that most of the antibiotic-resistant *E. coli* that humans carry probably comes from farm animals, especially chickens [4]. Because broiler manure is the main source of unmetabolized antibiotics in agricultural soil, It is important to investigate the extent of antibiotic contamination [5]. Based on market research, Fluoroquinolones, Lactams, Macrolides, Sulfonamides, and Tetracyclines are the top five antibiotics consumed in the animal industry [6], with Tetracyclines [7] and Fluoroquinolones being the most commonly applied antibiotics for broiler in China. The World Health Organization (WHO) also ranks Fluoroquinolones as an important veterinary antibiotic that may have an impact on human health [4]. As novel synthetic antibiotics of Tetracyclines and Fluoroquinolones, Doxycycline (DOX) and Gatifloxacin (GAT) are often used to prevent or treat diseases caused by Gram-positive and Gram-negative bacteria [8]. All broiler manure samples collected from ten different broiler farms in China were contaminated with at least seven antibiotics, whose concentration (DOX) could be as high as 78,516.1 μg/kg DW [5]. Unfortunately, no scientific information on residual GAT in broiler manure has been found so far, although GAT has already been used in the broiler industry in China to prevent and treat diseases. Similarly, the interaction of Fluoroquinolones with soil was highly specific, which suggested that broiler chicken production is a potential source of environmental pollution, but so far, the problem has been overlooked and needs to be further investigated [9].

The surge in demand and use of organic fertilizers necessitate more rigorous research to determine the safe limits of antibiotics in chicken manure for use in agricultural soil to reduce environmental pollution. Solid manure and waste water from animal husbandry are two major causes of environmental pollution. Anaerobic technology is often used to treat waste water [10,11], and aerobic composting is widely used to cycle nutrients, kill pathogens and stabilize organic matter in solid manure. Several studies have previously investigated the degradation of antibiotics during the composting process and have shown that composting is a successful method for degrading antibiotics in manure [12,13,14]. However, in some cases, certain antibiotics such as Sulfamethazine, Ofloxacin, and Ciprofloxacin are highly resistant to high-temperature composting and remain at high levels in composting products [15]. Berendsen et al. reported that the half-life of DOX during broiler manure composting was 20 days and that 49% of DOX still remained after 24 days of composting [7].

Apart from antibiotics, high levels of metal elements above the levels permitted by regulatory authorities such as the European Union and the National Research Council (NRC) are common in China. Metals in broiler manure can be extremely harmful to the environment if not properly treated because they tend to exist in active form [2]. A variety of metallic elements accumulate to varying degrees in the chicken manure, even after 85 days of co-composting with rice chaff [16]. The percentage of Zn and Cu increased by 112.8–192.7% and 115.5–132.6% after composting (60 days) [17]. Due to their high toxicity, persistence, and biological accumulation characteristics, many metal elements have become pollution in China’s agriculture [18].

A new study found that several new widely used antibiotics, such as DOX and GAT that have been frequently detected in waters [19]. However, their dissipation and persistence still remained unclear, especially the depletion and fate of GAT during the broiler composting process. Most of the studies were based on pig manure composting [20,21], and there was limited understanding of broiler manure composting. This study thus examined the accumulation of six metal elements and the fate and depletion of DOX and GAT during the composting process of broiler manure. The aim of this study is to determine the fate of heavy metals in broiler manure and the feasibility of removing DOX and GAT from broiler chicken manure during the composting process.

## 2. Materials and Methods

### 2.1. Chemicals and Standards

DOX premix (10% purity) and GAT premix (30% purity) were purchased from Borui biological engineering co. LTD, (Xi’an, Shanxi, China) and Huafei pharmaceutical co. LTD, (Shenyang, Liaoning, China), respectively. Premixed feed containing Fe: 10,000 mg/kg, Cu: 3200 mg/kg, Zn: 18,000 mg/kg, Mn: 20,000 mg/mg, Se: 60 mg/kg (recommended addition amount is 5 kg/T feed) was purchased from a feed company (An’shan, Liaoning, China). Premixed feed mainly provides animals with a variety of essential trace elements (Zn, Cu, Mn, etc.), but because its raw material source is often chemical industry waste water or minerals, it often contains a lot of heavy metals that are toxic and harmful to the environment.

### 2.2. Preparation and Collection of Broiler Manure

Five broiler chicken houses were randomly selected in a chicken farm in Liao Ning province, and 25,000 broilers were raised in each house. Each house as a treatment, five different feeding methods included: (1) 5 kg premix per ton feed (recommended amount) (T1), as control treatment, (2) 10 kg premix per ton feed (T2), (3) 15 kg premix per ton feed (T3), (4) 5 kg premix per ton feed, and therapeutic doses of DOX (31.2 mg/bird/day) and GAT (78 mg/bird/day) were given through drinking water (T4), (5) 5 kg premix per ton feed, and preventive doses of DOX (15.6 mg/bird/day) and GAT (48 mg/bird/day) were given through drinking water (T5). At the end of the normal feeding period (3 days), enough fresh broiler manure from each house was collected as composting materials.

### 2.3. Composting Experimental Design

The rice hull was purchased from a rice processing plant in LiaoNing province and pulverized to a size of 1–2 mm, and then uniformly mixed with each of five treatments of fresh broiler manure. The rice hull was added to adjust the initial C/N ratios to about 24. The primary components of the composting materials (including rice hull and five kinds of broiler manure) are shown in Table 1. The initial water content of these five treatments was adjusted to about 56%. No further water was added during the composting process. The compost fermenting agent (a commercial product purchased from Qiming Bio-Tech Co. Ltd., Yichang, Hubei, China) was mixed evenly at a rate equivalent to 0.1% of the compost weight. The five manure composting experiments (T1, T2, T3, T4 and T5) were conducted in triplicate using fifteen identical plastic barrels, each with a volume of 150 L. The composting system was connected to an 80-W powered pump to create a forced-aeration composting system. Aeration disc was placed at the bottom of each reactor to hold composting mixture and to promote air circulation. The aeration regularly was conducted for 20 min at 10:00 pm every day, and the manures were turned one time at 15:00–16:00 pm every day to promote uniform composting. The temperature was measured daily at 9:00 am and 15:00 pm using an automatic digital thermometer at the center of each material.

### 2.4. Sample Preparation

The composting process was stopped after 35 days when temperatures decreased to an ambient temperature level. Composite samples (1 kg) from days 1, 3, 5, 7, 10, 15, 20, 25, 30, and 35 of composting were collected with a scoop at different depths and corners after turning. The samples were divided into two parts and stored at −20 and −80 °C for chemical parameters analysis and detection of heavy metals and antibiotics, respectively.

### 2.5. Analytical Methods

The moisture content was determined by drying the samples at 105 °C to a constant weight. The pH was measured in a 1:10 (*w*/*v*, dry weight basis) water-soluble extract using a PHS-3C pH meter (INESA, Shanghai, China). The TN content was measured using the modified micro-Kjeldahl digestion method with an automatic FOSS-8100 Kjeldahl apparatus (FOSS, Hilleroed, Denmark). The OM and ash contents were measured with an SX-4-10 muffle furnace (Taiste, Tianjin, China) at 550 °C for 6 h. The total carbon (TC) content (%) was calculated from the ash fraction (%) with the following equation [22]:

TC(%)=(100%−ash%)/1.8

The contents of heavy metals were digested by using HNO_3_-HF-HClO_4_ (*v*/*v*/*v* 5:5:3) in the graphite furnace digestion system and measured using inductively coupled plasma optical emission spectrometry (Agilent Technologies 5100 ICP-OES, Santa Clara, CA, USA) and Atomic Fluorescence for Hg and As (PF-32, Persee, Beijing, China). Hg standard solution (100 μg/mL) (Aladdin, Shanghai, China) was used, and a multi-element mixed standard solution was used for the analysis of the other heavy metals (100 mg/L) (Macklin, Shanghai, China).

### 2.6. Modeling of Dissipation Kinetics

The dissipation of the target antibiotics was expressed as first-order kinetics:

(1) $$C(t)=C_{0}e^{\left(-\mathrm{kt}\right)}$$

where *C*_0_ (mg/kg) represents the initial analyte concentration of antibiotics, *C* is the analyte concentration (mg/kg) at time (*t*) (day of the experiment), and *k* is the antibiotic dissipation rate constant (day^−1^).

The half-life (*t*_1/2_) of the analytes is the time when the concentration reaches half of the initial concentration (*C*_0_), and it was calculated as follows:

(2) $$t_{1/2}=-\ln2/k$$

### 2.7. Statistical Analysis

Data analyses were performed using ANOVA of SPSS version 18.0 (SPSS for Windows) for the significance test. Redundancy analysis (RDA) was performed using Canoco 4.5 software [23] to analyze the correlations between the physicochemical properties and heavy metals and antibiotics among the five treatments during composting.

## 3. Results

### 3.1. Persistence of Antibiotics (DOX and GAT) during the Composting Process

**Figure 1:**
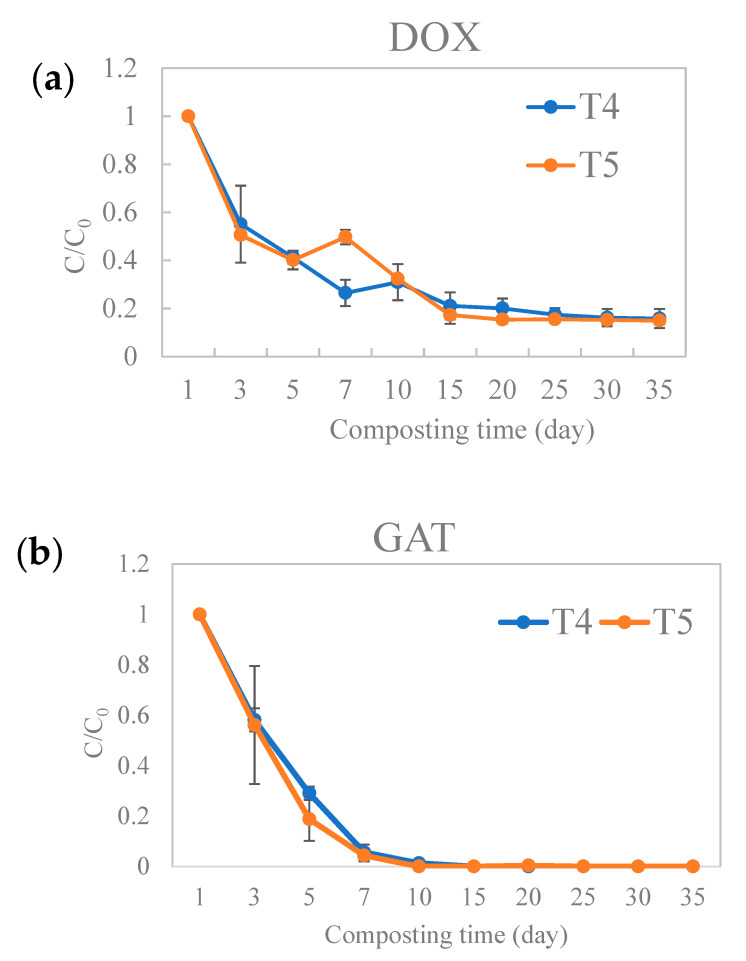
Changes in the antibiotics relative content in each group (T4 and T5). Abbreviations: (**a**) DOX, Doxycycline; (**b**) GAT, Gatifloxacin.

The fates of antibiotics during composting are shown in Figure 1. After 35 days of composting, the total DOX removals were 84.16% (T4) and 85.04% (T5), respectively. The DOX levels of both T4 and T5 decreased rapidly, from 137 mg/kg DW to 42.4 mg/kg DW (T4) and from 72.2 mg/kg DW to 23.4 mg/kg DW (T5) after 10 days of composting. After rapid removal of DOX (69.05% for T4 and 67.59% for T5) during the thermophilic period (i.e., the first seven days), its concentration slowly decreased and stabilized. At the end of the composting period, only about 21.7 mg/kg and 10.8 mg/kg of DOX in T4 and T5, representing 15.84% and 14.96% of the initial concentrations, remained in the composting masses. In contrast to DOX, the GAT was almost completely removed (T4: 98.66%, T5: 100%) after 10 days of composting, indicating the biodegradation of GAT was easier than that of DOX (Figure 1b). The removal rate was especially high (94.36% and 95.65% for T4 and T5, respectively) during the first 7 days of the thermophilic stage, which then dropped to only 4.3% and 4.35% between the 7th and the 10th day.

### 3.2. Dissipation Kinetics of DOX and GAT during Broiler Manure Composting

The removal of DOX and GAT was modeled using the first-order equation to obtain the conjugate reaction formulas. The results indicated that the dissipation of DOX and GAT during the broiler manure composting process could be expressed with first-order kinetics. The regression coefficients (*R*^2^) of all the treatments (T4, T5) ranged from 0.7452 to 0.9703 (Table 2). The natural logarithm of the concentration was divided by the starting concentration (ln *C*/*C*_0_) versus time (*t*) plot shows a straight line with a good regression for all treatment, indicating that the degradation of DOX and GAT in this study followed the first-order depletion. The half-life (*t*_1/2_) of the DOX and GAT, calculated according to Fitting Equation (2) based on the slope of the straight line, ranged from 1.32 to 15.86 days with a rate constant of −0.0437/day to −0.5251/day (Table 2).

The results showed that there were different half-lives of DOX and GAT depletion in the two treatments with the same manure composting condition. The depletion of GAT was faster than that of DOX in broiler manure composting, but the half-life of the two antibiotics in T4 treatment was slightly higher than that in T5 treatment.

### 3.3. Changes in the Total Amount of Heavy Metals during Composting

Changes in the contents of some major heavy metal elements are shown in Table 3. The contents of most heavy metals in three treatments (T1, T2, and T3) increased gradually from the initial phase to the end of the composting process, while the concentrations of Hg varied throughout the whole composting process. In this study, the concentration of Cr, Cu, Pb, Zn, and As in manure also increased with the increase in premix addition.

### 3.4. Redundancy Analysis (RDA) of Antibiotics and Heavy Metals Levels and Physicochemical Properties during Composting

**Figure 2:**
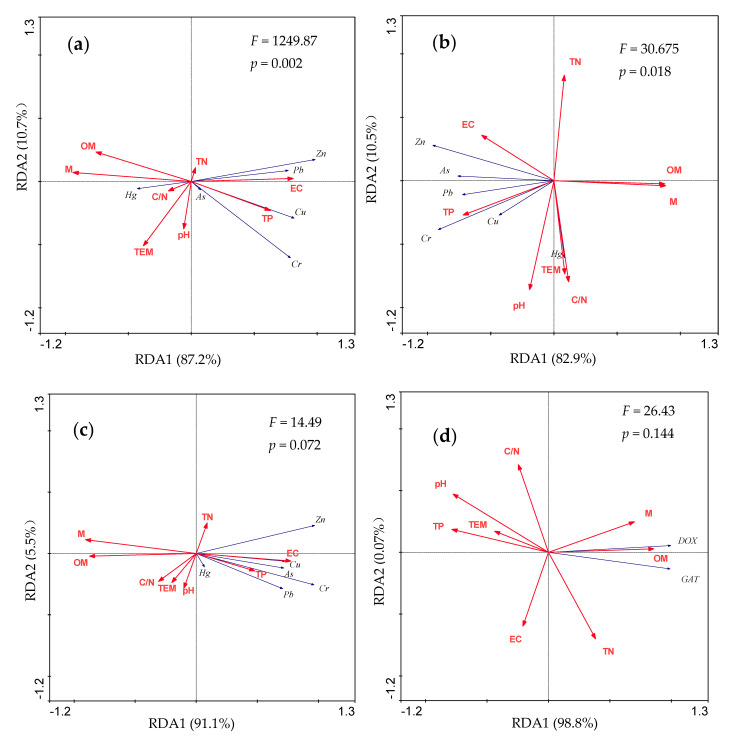

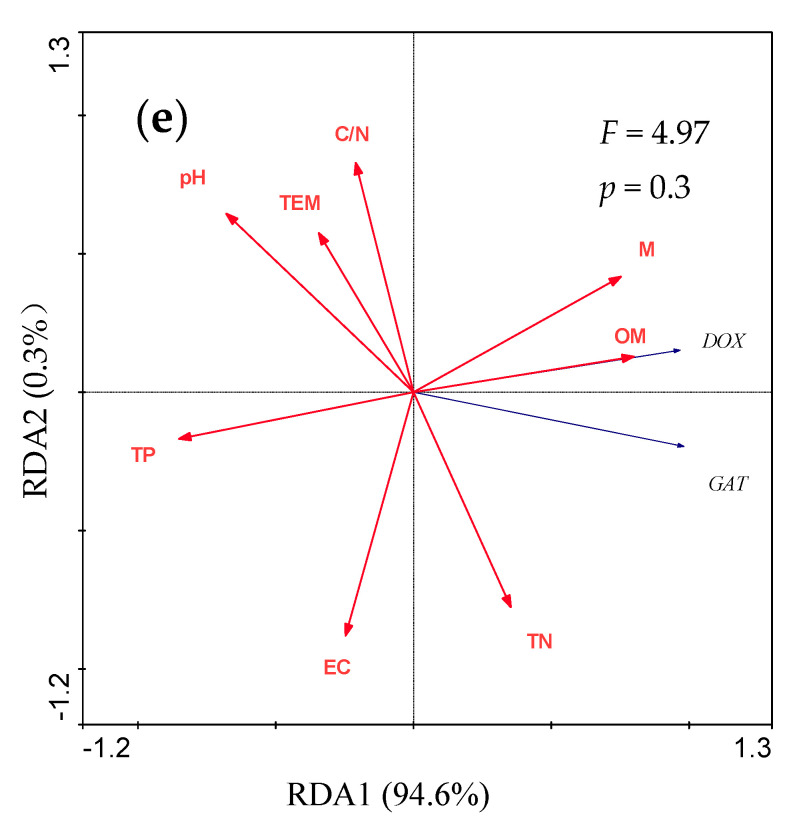
Redundancy analysis (RDA) assessing the relationship between environmental factors (red arrows) and antibiotics/metals (blue arrows). (**a**) T1; (**b**) T2; (**c**) T3; (**d**) T4; (**e**) T5. Abbreviations: TEM, temperature; M, moisture.

The relationships between antibiotic levels and environmental factors of the composting process of broiler manure were determined based on the Redundancy analysis (RDA) (Figure 2). The length and direction of the arrows in the figures reflect the importance and correlation of the variables. The results indicated that moisture, OM, TP, temperature, and pH were positively correlated with the first axis (explaining 99.8% of the total variance in Figure 2d and 94.6% of the total variance in Figure 2e), with C/N, EC, and TN positively correlated with the second axis (explaining 0.7% of the total variance in Figure 2d and 3% of the total variance in Figure 2e).

The degradation of DOX and GAT were positively correlated with OM and moisture, such as DOX with OM (r = 0.86) and moisture (r = 0.71) in T4 treatment, DOX with OM (r = 0.79) and moisture (r = 0.79) in T5. The concentrations of targeted antibiotics were negatively correlated with pH and TP.

Meanwhile, the variation trend of all metal content in the composting process was significantly positively correlated with TP and EC, while negatively correlated with moisture and OM, except for Hg (Figure 2).

## 4. Discussion

Collectively, temperature, moisture, pH, EC, TN, OM, C/N, and TP content data suggest that the results of the composting process are in full compliance with organic manure standards (55 °C for 5 days, pH 6–9), and the composting intensity was relatively similar between the control and the other treatments. However, the composting intensity was slightly different at the initial stage (T1, T2, T3 > T4, T5), indicated by the parameters of the composting mass among the five treatments in most cases (Table S1, see Supplementary Materials). This was expected since DOX and GAT concentrations in manure had an important effect on microbial activities in the composting process. However, the concentration of metals did not show a notable stimulative or suppressive effect through the whole composting process.

This study showed the effectiveness of composting in eliminating antibiotics in broiler manure, like in other studies, which also presented a decreasing antibiotic level under various experimental conditions [13,14]. The efficiencies of antibiotic removal vary significantly due to experimental design and the types of antibiotics, ranging from 54% for Monensin in turkey manure [24] to more than 99% for Erythromycin, Salinomycin and Trimethoprim in poultry manure [13]. The removal can even reach 100% for Sulfadiazine in pig manure [14]. The addition of other organic materials such as rice straw [25] or sawdust [26] as compost mixture provides many adsorption points for antibiotic removal.

According to previous data, Tetracyclines tested in a single type of manure (type of manure not indicated) are very persistent [27]. A possible explanation could be that a large fraction of the Tetracyclines instantaneously binds to solid particles and that, as a consequence, only part is available for the dissipation processes, which has previously been suggested to be an enzymatic process. The strong binding delays the biodegradation [28], and thus, Tetracyclines can persist in manure for a long time. However, another study showed that antibiotics from the group of Tetracyclines are unstable because of their unique chemical structure and may undergo abiotic degradation conditions, such as pH, temperature, redox, and light conditions, and then generate degradation products through epimerization, dehydration, or other pathways [29]. High-temperature composting is an effective way to eliminate Tetracyclines in manures as they are very sensitive to temperature [30]. For example, Tetracyclines were eliminated to a higher extent at 55 °C than at 22 °C and 38 °C within compost treatments [31]. However, further research on its mechanism requires more experiments in sterile reactors because microbes are often important indicators of the process of aerobic and anaerobic reactions [32].

Drugs from the Tetracyclines family vary in their lipid solubility, and this property largely influences their absorption, distribution, and excretion pattern. The newer Tetracyclines, such as DOX and Minocycline, are more lipid soluble than the older derivatives. Consequently, their pharmacokinetics differs greatly from that of the older Tetracyclines (Oxytetracycline, Chlortetracycline and Tetracycline) [33]. Ho et al. reported a removal efficiency of about 99.8% from the initial concentration 13.63 mg/kg to the final concentration 0.027 mg/kg of DOX content [13]. However, DOX in this experiment showed a lower removal rate than other Tetracycline antibiotics, such as 87.8% for Oxytetracycline [12] and nearly 100% for Chlortetracycline [14]. This study also showed that DOX degradation mainly occurred in the early stage of the thermophilic phase (before 10 days) but was inhibited after day 15. About 15% of DOX cannot be degraded after the end of composting, regardless of the initial DOX concentration. A simulation test also showed that DOX cannot be removed completely despite the long-term composting process [34]. In the current study, the half-life of DOX was 13.75 and 15.86 days (Table 2), which was rather different from that of 3.8 days during composting of poultry manure reported by Ho et al. [13]. It indicates that DOX is not innately degradable, the half-life reported for DOX was heterogeneous, but its dissipation depends on specific conditions employed in the respective experiment [35]. Other processes than composting may therefore be required if we want to achieve 100% DOX removal [36]. By comparing the nodes where DOX stopped degrading with the nodes where the temperature dropped, we suspected that low temperature might be responsible for DOX persistence. Moreover, the higher pH value will lead to the higher response of microorganisms to the external environment, and the more obvious their biological activity [37]. The above inferences are basically consistent with the results of RDA analysis in this study (Figure 2). Therefore, increasing the temperature of composting and extending the high temperature time or increasing the pH value of the pile should be both effective methods to improve the degradation rate of antibiotics.

In contrast to DOX, GAT could be completely degraded in this study. So far, the mechanism for GAT degradation is not fully understood. A study showed that adsorption, not biodegradation, is the primary removal mechanism of Fluoroquinolones compounds [38]. The rapid dissipation of GAT could therefore be attributed to the electrophilicity of its hydroxyl group to reduce its distribution in the adsorption phase [39]. Therefore, the easy degradation characteristic of GAT in the composting process may be due to its unique chemical properties, leading to its different degradation trend from other Fluoroquinolones.

Because this was the first time GAT degradation was analyzed, we only compared our results with the results of other Fluoroquinolones. Other Fluoroquinolones do not change during animal excretion and may undergo conjugation, oxidation, hydroxylation, dealkylation (for example, Enroxacin is de-ethylated to Ciprofloxacin) or decarboxylation. A significant number of compounds (most Fluoroquinolones are not metabolized) and their metabolites may enter the soil through urine and feces. Similar to other antibiotics, photochemical degradation is one main pathway for the Fluoroquinolones to degrade in the environment [40]. After irradiation, the quinolone ring remains generally intact, despite chemical changes in structure, so that its antimicrobial activity remains generally high. Even small amounts of the product enter the environment and persist, which can lead to microbial resistance [2]. However, not all the results of the composting experiment on Fluoroquinolones antibiotics agree with the above conclusions. More than 99% of Enrofloxacin, Flumequine, and Norfloxacin involved were removed from the broiler manure during 40 days of composting, which showed a short half-life in broiler manure composting, ranging from 1.3 to 3.8 days [13].

Information about the fate of Fluoroquinolones during the composting process is limited. Unlike the results of this study, the half-life of Norfloxacin and Lomefloxacin should be18.0 days and 32.5 days [41] in broiler manure composting, which were significantly higher than those presented by Ho et al. [13], who reports a *t*_1/2_ of 2.8 days for Enrofloxacin and 2.1 days for Norfloxacin. By comparison, it was found that the half-life of GAT observed in this experiment was very close to the 1.3 days half-life of Flumequine reported by Selvam et al. [14]. However, the *t*_1/2_ of Ciprofloxacin was 15.8–20.8 days during pig manure composting, and only 60–83% of the Ciprofloxacin was degraded after 56 days of composting [14]. This may be related to the initial inhibition in the composting process, which the high residual concentration with 1 mg/kg initial concentration could be due to the sorption and nonextractability of the compound. These results confirmed a relatively rapid removal for the target GAT compared to the other fluoroquinolones. It also showed that GAT, a novel antibiotic, is significantly different from most Fluoroquinolones.

According to the degradation trend of DOX and GAT of T4 and T5 in this study, we can draw the following conclusions: (1) under the same experimental conditions, the degradation rate and half-life of antibiotics are determined by the type of antibiotics, and the half-life difference of different types of antibiotics is huge. (2) For the same antibiotics, the amount of antibiotics in manure has a certain effect on the half-life of antibiotics in the composting process, but it is not significant.

Table 3 showed the increasing concentration of various metals during the composting process, similar to some studies [42,43]. Ravindran et al., for example, reported that after 50 days of composting, the concentration of Cu and Zn in pig manure increased compared with the initial stage [42]. Metals become concentrated as the decomposition of OM releases gases (CO_2_ and NH_3_), and moisture evaporates. Our results also showed that increasing the use of premix feed (T2: 10 kg/T and T3: 15 kg/T) increased the concentrations of Cu, Cr, Zn, Pb, and As in manure. Because all heavy metals are non-biodegradable and tend to be concentrated during composting, applying manure for agricultural soil may pose a potential threat to animal and human health through the food chain. Limiting the use of heavy metal elements in the livestock industry should therefore become the first measure to reduce soil metal pollution.

Different from other metals, the concentrations of Hg varied throughout the whole composting process. It is possible that that the content of Hg in the raw materials used in the agricultural and animal husbandry industries in northeast China might be too low to be detected by the Atomic Fluorescence Spectrometer. Another possible reason is that the composting process experienced constant high temperature and ventilation, and Hg is likely to disperse with the airflow. We suggested that Hg could be lost through evaporation because there was no correlation between Hg and environmental factors. This finding is in contrast with a significantly negative correlation between other heavy metals with moisture and OM (Figure 2a–c).

Heavy metal contents are key parameters in composting, especially when the compost is used as a source of organic fertilizer. According to the American Ecological criteria [44], the final contents of Cu, As, Pb and Hg in the organic fertilizers prepared in this experiment have no environmental hazards for soil application, even if the use of premix feed is appropriately increased. However, it is necessary to pay attention to the accumulation of Zn and Cr in the manure of broiler chicken. Because these two kinds of metal contents were higher than the limits allowed by the American and Ecological criteria (i.e., 300 mg/kg D.W and 100 mg/kg D.W in composts for Zn and Cr, respectively). Yet this study showed that the contents of Zn reach 258.2 mg/kg (T1), 312.21 mg/kg (T2), and 333.68 mg/kg (T3) after 35 days of composting, and that of Cr reached 160.16 mg/kg (T1), 223.98 mg/kg (T2), and 248.02 mg/kg (T3). Adding Cr and Zn in the broiler premix feed can therefore cause an environmental concern, particularly if the manure is applied to agricultural soils.

## 5. Conclusions

Our results showed that DOX might pose a greater risk to the environment in comparison to GAT. Even after the standard aerobic composter process, 14.96–15.84% of DOX residues remained, in comparison to no GAT residues. Based on the RDA, the concentration of antibiotics was suggested to be significantly influenced by physico-chemical properties (mainly temperature and pH) throughout the composting process. Therefore, increasing the temperature of composting and extending the high-temperature time or increasing the pH value of the pile should be both effective methods to improve the degradation rate of antibiotics. Our results also showed that the metal concentration increased with the amount of trace elements in the feed materials, which could be passed on to humans when manure is used for organic fertilizer. In this study, the Cr content in T1, T2, and T3 treatments reached 160.16 mg/kg, 223.98 mg/kg, and 248.02 mg/kg after 35 days of composting, while Zn in T2 and T3 reached 312.21 mg/kg and 333.68 mg/kg, respectively. These numbers exceed the United States and the European soil requirements. China should, therefore, regulate the amount of trace metals in feed materials to reduce potential risks to the soil ecosystem.

## Figures and Tables

**Table 1:** Characteristics of raw materials for broiler manure composting.

Characteristics	Broiler Manure					Rice Hull
	T1	T2	T3	T4	T5	
Moisture content (%)	78.44 ± 0.69	77.34 ± 0.89	77.46 ± 1.61	78.3 ± 0.39	79.97 ± 1.35	12.59 ± 0.23
pH	5.66 ± 0.18	5.58 ± 0.21	5.73 ± 0.24	5.84 ± 0.07	5.62 ± 0.09	6.86 ± 0.33
EC (us/cm)	590 ± 24.15	626 ± 32.93	608 ± 27.58	617 ± 19.60	604 ± 9.43	ND
TN (%)	5.05 ± 0.02	4.55 ± 0.01	4.54 ± 0.17	4.52 ± 0.07	4.62 ± 0.04	0.42 ± 0.01
C/N	9.47 ± 0.05	10.57 ± 0.02	10.61 ± 0.41	10.63 ± 0.34	10.38 ± 0.21	111.39 ± 3.26
TP (%)	1.16 ± 0.04	1.05 ± 0.01	1.11 ± 0.07	1.12 ± 0.01	1.05 ± 0.02	0.09 ± 0.01
OM (%)	86.21 ± 0.11	86.56 ± 0.08	86.67 ± 0.19	86.52 ± 0.22	86.33 ± 0.23	84.21 ± 0.15
Cu (mg/kg)	60.29 ± 1.19	77.4 ± 0.3	78.09 ± 0.75			30.59 ± 4.03
Zn (mg/kg)	500.26 ± 12.16	644.65 ± 10.25	655.81 ± 12.02			63.2 ± 1.56
Pb (mg/kg)	0.62 ± 0.03	0.78 ± 0.04	0.83 ± 0.27			0
As(mg/kg)	0.22 ± 0.01	0.27 ± 0.02	0.32 ± 0.02			0.66 ± 0.03
Hg (mg/kg)	0.005 ± 0.001	0.005 ± 0.002	0.004 ± 0.003			0
Ni (mg/kg)	3.02 ± 0.01	3.46 ± 0.37	4.02 ± 0.33			1.33 ± 0.16
Cr (mg/kg)	218.13 ± 19.53	398.01 ± 6.36	418.94 ± 1.88			26.51 ± 2.60
Concentration of DOX (mg/kg)				250.4 ± 3.2	143.2 ± 7.2	
Concentration of GAT (mg/kg)				185.9 ± 13.97	146.8 ± 10.1	

Measured base on a dry weight basis. ND means not detected. Abbreviations: EC, electrical conductivity; OM, organic matter; TN, total N; TP, total phosphorus.

**Table 2:** The fitting equation, slope of linear regression (k), regression coefficient (R^2^), and the half-life (*t*_1/2_) of DOX and GAT in different treatments.

Antibiotics	Treatments	Fitting Equation	Slope (*k*)	*R* ^2^	Half-Life (*t*_1/2_) (Days)
GAT	T4	ln *C/C*_0_ = −0.5011X + 0.812	−0.5011	0.9703	1.38
	T5	ln *C/C*_0_ = −0.5251X + 0.7539	−0.5251	0.9655	1.32
DOX	T4	ln *C/C*_0_ = −0.0437X − 0.5954	−0.0437	0.7452	15.86
	T5	ln *C/C*_0_ = −0.0504X − 0.5085	−0.0504	0.7812	13.75

**Table 3:** Changes in major heavy metal elements (Cu, Zn, Cr, As, Pb, Hg) during the composting process. The results are expressed as the mean plus standard error of three replicates.

Time (Day)	Cu mg/kg			Zn mg/kg			Cr mg/kg		
	T1	T2	T3	T1	T2	T3	T1	T2	T3
1	25.18 ± 1.62	34.43 ± 0.81	34.49 ± 1.39	204.92 ± 5.23	249.77 ± 0.42	262.74 ± 7.28	119.53 ± 10.01	172.29 ± 15.08	195.29 ± 21.59
3	28.37 ± 1.15	33.48 ± 0.48	36.49 ± 0.88	220.21 ± 4.10	271.96 ± 9.05	283.25 ± 10.96	150.54 ± 6.30	208.20 ± 17.40	224.33 ± 9.82
5	30.04 ± 0.07	35.25 ± 3.04	37.11 ± 1.11	196.79 ± 14.14	256.44 ± 11.74	274.04 ± 7.14	129.04 ± 1.43	212.07 ± 7.23	216.96 ± 1.65
7	27.32 ± 3.72	33.17 ± 2.36	35.39 ± 0.16	199.25 ± 6.58	257.47 ± 8.77	272.58 ± 2.33	137.38 ± 3.22	203.11 ± 4.11	206.76 ± 15.46
10	28.66 ± 0.48	58.15 ± 3.61	50.19 ± 0.81	210.45 ± 7.92	261.67 ± 10.11	270.84 ± 3.96	143.2 ± 7.14	212.4 ± 1.77	207.42 ± 1.03
15	32.59 ± 3.79	47.5 ± 4.95	55.83 ± 1.34	245.34 ± 13.19	273.21 ± 20.77	289.98 ± 17.06	134.52 ± 0.80	219.44 ± 8.66	237.66 ± 16.92
20	45.67 ± 2.71	38.84 ± 0.72	44.48 ± 1.03	232.09 ± 5.29	298.09 ± 13.62	302.29 ± 18.37	152.33 ± 5.93	238.55 ± 10.17	246.87 ± 4.05
25	37.45 ± 3.43	45.34 ± 3.24	47.34 ± 4.04	257.56 ± 15.30	277.55 ± 7.94	288.79 ± 23.32	147.87 ± 6.69	221.76 ± 6.02	243.99 ± 5.52
30	43.66 ± 0.83	46.97 ± 1.28	53.66 ± 0.36	244.53 ± 6.32	301.39 ± 7.40	319.62 ± 5.61	158.52 ± 2.35	228.39 ± 11.10	257.88 ± 8.84
35	42.81 ± 2.18	47.86 ± 0.78	52.78 ± 2.68	258.20 ± 3.57	312.21 ± 12.10	333.68 ± 9.62	160.16 ± 3.24	223.98 ± 5.68	248.02 ± 7.05
*p*	<0.01	<0.01	<0.01	<0.01	<0.01	<0.01	<0.01	<0.01	<0.01
**Time** **(Day)**	**As mg/kg**			**Pb mg/kg**			**Hg mg/kg**		
	**T1**	**T2**	**T3**	**T1**	**T2**	**T3**	**T1**	**T2**	**T3**
1	0.608 ± 0.02	0.558 ± 0.01	0.611 ± 0.07	0.215 ± 0.03	0.247 ± 0.06	0.29 ± 0.13	0.008 ± 0.003	0.011 ± 0.001	0.012 ± 0.003
3	0.568 ± 0.01	0.513 ± 0.13	0.643 ± 0.02	0.276 ± 0.18	0.221 ± 0.17	0.345 ± 0.06	0.011 ± 0	0.018 ± 0.001	0.012 ± 0.001
5	0.621 ± 0.03	0.574 ± 0.04	0.587 ± 0.01	0.356 ± 0.19	0.429 ± 0.08	0.478 ± 0.05	0.046 ± 0.001	0.029 ± 0.008	0.028 ± 0.005
7	0.849 ± 0.21	0.632 ± 0.06	0.628 ± 0.05	0.317 ± 0.12	0.505 ± 0.13	0.390 ± 0.13	0.026 ± 0.001	0.014 ± 0.002	0.034 ± 0.01
10	0.569 ± 0.04	0.545 ± 0.02	0.665 ± 0.03	0.315 ± 0.02	0.344 ± 0.18	0.376 ± 0.01	0.01 ± 0.002	0.017 ± 0.004	0.011 ± 0.002
15	0.634 ± 0.11	0.667 ± 0.01	0.81 ± 0.19	0.565 ± 0.07	0.515 ± 0.24	0.436 ± 0.04	0.016 ± 0.003	0.03 ± 0.01	0.015 ± 0
20	0.597 ± 0.06	0.768 ± 0.22	0.673 ± 0.01	0.448 ± 0.15	0.537 ± 0.04	0.599 ± 0.03	0.018 ± 0.01	0.021 ± 0.01	0.024 ± 0.008
25	0.692 ± 0.03	0.643 ± 0.01	0.742 ± 0.12	0.421 ± 0.02	0.479 ± 0.05	0.766 ± 0.02	0.013 ± 0.001	0.012 ± 0.002	0.013 ± 0.001
30	0.702 ± 0.15	0.681 ± 0.01	0.725 ± 0.04	0.517 ± 0.06	0.567 ± 0.10	0.521 ± 0.17	0.008 ± 0.003	0.011 ± 0.001	0.026 ± 0.004
35	0.699 ± 0.09	0.698 ± 0.12	0.758 ± 0.01	0.495 ± 0.11	0.575 ± 0.02	0.650 ± 0.09	0.021 ± 0.012	0.010 ± 0.001	0.013 ± 0.001
*p*	<0.01	<0.01	<0.01	<0.01	<0.01	<0.01	<0.01	<0.01	<0.01

Measured base on a dry weight basis.

## Data Availability

All data are available in this manuscript.

## Supplementary Materials

The following are available online. Table S1: Changes of Temperature, TN, TP, EC, PH, Moisture, OM and C/N during composting.

## Sample Availability

Samples of the compounds are available from the authors.
