# Supplementary material for: Dissipation Dynamics of Doxycycline and Gatifloxacin and Accumulation of Heavy Metals during Broiler Manure Aerobic Composting
Source: Molecules. 2021 Aug 28;26(17):5225. doi: 10.3390/molecules26175225 (PMC8434052; doi:10.3390/molecules26175225)
Supplement: Supplementary file 1 [file molecules-26-05225-s001.zip › molecules-1296056-supplementary.pdf]

Table S1 Changes of Temperature, TN, TP, EC, PH, Moisture, OM and C/N during composting.

Time (day)	Temperature (°C)				
	T1	T2	T3	T4	T5
1	13.5±0.75	12.3±0.59	12.7±0.72	12.4±0.86	13.1±0.56
3	57.0±1.29	54.0±0.70	55.0±1.11	45.5±1.01	48.3±1.18
5	63.1±0.93	64.7±0.68	62.5±0.86	60.1±1.19	61.8±2.16
7	60.1±2.07	58.3±0.68	57.5±0.98	60.4±1.08	57.4±1.21
10	57.2±0.82	55.8±1.88	56.5±0.87	54.3±0.91	54.9±0.64
15	40.3±2.50	45.1±1.21	45.3±1.19	43.9±1.08	41.4±0.56
20	42.6±1.11	38.4±1.07	40.8±0.79	39.7±1.36	36.8±0.56
25	35.2±0.53	36.9±0.55	37.7±1.23	34.2±0.47	33.5±1.74
30	31.5±1.46	32.1±1.15	35.9±0.53	36.4±0.56	35.7±1.15
35	33.5±0.92	33.2±0.74	32.5±0.95	30.6±2.50	31.4±0.36
p	<0.01	<0.01	<0.01	<0.01	<0.01
Time (day)	TN (%)				
	T1	T2	T3	T4	T5
1	2.04±0.07	1.96±0.12	1.93±0.02	1.82±0.03	1.87±0.10
3	1.95±0.03	1.81±0.07	1.97±0.03	1.83±0.03	1.86±0.04
5	1.56±0.15	1.55±0.03	1.64±0.03	1.74±0.04	1.82±0.07
7	1.37±0.14	1.35±0.02	1.35±0.02	1.54±0.01	1.22±0.10
10	1.32±0.11	1.21±0.01	1.32±0.06	1.34±0.05	1.28±0.08
15	1.36±0.01	1.36±0.02	1.36±0.03	1.33±0.02	1.26±0.03
20	1.33±0.09	1.41±0.02	1.48±0.02	1.38±0.04	1.55±0.03
25	1.59±0.02	1.65±0.03	1.63±0.14	1.74±0.04	1.69±0.12
30	1.75±0.01	1.72±0.05	1.77±0.10	1.79±0.02	1.81±0.06
35	1.81±0.03	1.78±0.03	1.75±0.06	1.87±0.08	1.82±0.05
p	<0.01	<0.01	<0.01	<0.01	<0.01
Time (day)	TP (%)				
	T1	T2	T3	T4	T5
1	0.398±0.007	0.393±0.011	0.414±0.003	0.457±0.033	0.424±0.021
3	0.464±0.053	0.421±0.008	0.490±0.021	0.450±0.014	0.438±0.015
5	0.493±0.028	0.482±0.014	0.546±0.028	0.452±0.025	0.450±0.007

7	0.533±0.049	0.495±0.005	0.548±0.005	0.500±0.008	0.489±0.067
10	0.519±0.007	0.490±0.021	0.543±0.014	0.537±0.003	0.523±0.005
15	0.492±0.028	0.487±0.007	0.512±0.010	0.531±0.017	0.514±0.007
20	0.470±0.007	0.482±0.078	0.519±0.011	0.564±0.019	0.542±0.030
25	0.589±0.014	0.543±0.042	0.573±0.007	0.537±0.016	0.571±0.030
30	0.580±0.026	0.530±0.009	0.540±0.008	0.560±0.006	0.560±0.003
35	0.560±0.037	0.540±0.010	0.560±0.003	0.550±0.005	0.570±0.006
p	<0.01	<0.01	<0.01	<0.01	<0.01

Time (day)	EC (us·cm^{-1})				
	T1	T2	T3	T4	T5
1	703±24.04	721.5±38.69	727.5±16.26	733.4±8.67	719±1.61
3	657±11.31	698±19.50	621.5±9.19	715.3±22.43	702.1±17.62
5	606.5±3.54	648±21.21	634±12.73	701.1±13.85	687.5±2.73
7	608±4.24	598±43.34	598.5±10.61	613.9±7.58	639.2±15.45
10	634.5±14.85	617.5±0.71	590.5±9.19	604±9.32	598.1±6.81
15	749±8.49	793±36.27	747.5±7.78	777.4±24.69	724.3±18.01
20	831.5±41.42	722.5±23.33	806±4.24	756.96±11.44	788.9±14.10
25	733±38.08	852.5±40.61	781.5±36.06	818.32±33.30	757.7±37.96
30	815±26.27	786±32.17	794.9±11.75	779.6±3.46	764.3±31.03
35	803±13.75	837±6.48	816±6.20	846.3±25.20	777.3±19.36
p	<0.01	<0.01	<0.01	<0.01	<0.01

Time (day)	pH				
	T1	T2	T3	T4	T5
1	5.77±0.00	5.63±0.23	5.67±0.06	5.82±0.07	5.74±0.32
3	7.07±0.28	6.61±0.06	6.92±0.48	6.34±0.37	6.67±0.10
5	7.64±0.09	6.97±0.22	7.18±0.08	6.88±0.07	7.1±0.27
7	7.44±0.17	7.49±0.36	7.70±0.07	7.21±0.10	7.54±0.18
10	7.18±0.12	7.34±0.14	7.32±0.04	7.59±0.10	7.35±0.05
15	7.10±0.05	7.14±0.06	7.15±0.06	7.23±0.24	7.18±0.05
20	6.91±0.04	6.99±0.09	6.82±0.16	6.75±0.08	6.85±0.09
25	7.09±0.10	7.09±0.06	6.87±0.08	7.12±0.05	6.73±0.07
30	6.93±0.08	6.70±0.28	6.64±0.37	6.88±0.18	6.77±0.24
35	6.74±0.10	6.40±0.18	6.51±0.06	6.66±0.32	6.85±0.17
p	<0.01	<0.01	<0.01	<0.01	<0.01

Time (day)	Moisture (%)				
	T1	T2	T3	T4	T5

1	55.4±3.65	56.02±2.65	56.4±0.94	57.3±0.71	56.9±0.48
3	55.23±1.11	54.73±0.68	55.63±0.19	56.82±1.68	56.75±0.52
5	56.6±0.37	55.2±0.45	56.18±0.43	56.98±2.28	55.48±3.22
7	54.18±1.77	52.84±0.75	54.93±1.07	55.23±3.56	53.75±2.36
10	52.22±4.25	49.72±1.68	50.69±2.18	53.64±1.65	53.59±1.89
15	49.57±3.39	44.52±3.00	46.57±2.71	50.19±0.44	47.86±1.36
20	46.98±2.37	43.61±0.59	44.66±3.34	47.37±2.10	47.43±2.46
25	44.69±1.23	41.81±1.56	41.9±2.18	43.89±3.83	42.74±2.42
30	43.5±0.92	41.2±3.92	40.3±0.83	44.58±1.98	42.83±1.70
35	41.6±1.89	39.3±1.37	40.4±1.67	42.09±0.59	43.1±0.89
p	<0.01	<0.01	<0.01	<0.01	<0.01
Time (day)	OM (%)				
	T1	T2	T3	T4	T5
1	84.01±0.10	84.26±0.12	84.01±0.10	84.26±0.05	83.15±0.29
3	83.11±0.10	83.32±0.06	83.09±0.01	83.64±0.01	82.84±0.35
5	81.67±0.05	82.31±0.09	82.02±0.19	82.77±0.02	82.03±0.13
7	80.73±0.43	81.54±0.17	81.16±0.01	81.64±0.35	80.77±0.90
10	80.87±0.22	81.03±0.14	80.49±0.06	80.77±0.47	79.76±0.04
15	80.53±0.15	80.34±0.14	80.01±0.05	80.63±0.00	80.13±0.27
20	80.39±0.13	80.09±0.25	79.54±0.29	80.09±0.06	79.63±0.30
25	79.90±0.25	79.26±0.35	78.92±0.12	80.06±0.05	79.32±0.60
30	79.00±0.09	78.20±0.04	78.01±0.14	78.80±0.13	77.70±0.25
35	77.50±0.22	77.00±0.30	77.20±0.25	78.20±0.27	76.80±0.41
p	<0.01	<0.01	<0.01	<0.01	<0.01
Time (day)	C/N				
	T1	T2	T3	T4	T5
1	22.91±0.75	23.89±1.55	24.23±0.24	25.73±0.42	24.70±0.22
3	23.69±0.40	25.51±1.03	23.44±0.40	25.40±0.45	24.74±0.36
5	29.06±2.82	29.51±0.57	27.82±0.54	26.43±0.59	25.05±0.91
7	32.70±3.87	33.62±0.48	33.42±0.53	29.45±0.12	36.81±0.91
10	33.98±2.93	37.07±0.18	33.98±1.54	33.53±1.44	34.61±2.03
15	32.89±0.12	32.89±0.58	32.69±0.75	33.69±0.48	35.34±0.64
20	33.51±2.25	31.67±0.59	29.79±0.32	32.25±0.80	28.55±0.41
25	27.86±0.34	26.65±0.29	26.96±2.23	25.53±0.51	26.10±1.57
30	25.08±0.13	25.26±0.13	24.52±1.28	24.45±0.22	23.86±0.71
35	23.83±0.28	24.01±0.31	24.50±0.79	23.26±0.22	23.45±0.46

p	<0.01	<0.01	<0.01	<0.01	<0.01
